# 2042 CYP2C19*2 and PON1 Q192R polymorphisms are associated with platelet reactivity to clopidogrel in Puerto Rican Hispanics with cardiovascular disease

**DOI:** 10.1017/cts.2018.58

**Published:** 2018-11-21

**Authors:** Dagmar F. H. Suarez, Mariana R. Botton, Stuart A. Scott, Matthew I. Tomey, Kyle Melin, Angel Lopez-Candales, Jessicca Y. Renta, Jorge Duconge

**Affiliations:** Icahn School of Medicine at Mount Sinai

## Abstract

OBJECTIVES/SPECIFIC AIMS: High on-treatment platelet reactivity (HTPR) with clopidogrel imparts an increased risk for ischemic events in adults with coronary artery disease. Although more potent antiplatelet agents are available, clopidogrel remains the most commonly used P2Y12 inhibitor in Puerto Rico. Platelet reactivity varies with ethnicity and is influenced by both clinical and genetic variables; however, no clopidogrel pharmacogenetic studies with Puerto Rican patients have been reported. Therefore, we sought to identify clinical and genetic determinants of on-treatment platelet reactivity in a cohort of Puerto Rican patients with cardiovascular disease. METHODS/STUDY POPULATION: We performed a retrospective study of 111 Puerto Rican patients on 75 mg/day maintenance dose of clopidogrel. Patients were allocated into 2 groups: Group I, without HTPR; and Group II, with HTPR. Clinical data was obtained from the medical record. Platelet function was measured ex vivo using the VerifyNow^®^ P2Y12 assay and HTPR was defined as P2Y12 reaction units (PRU)≥230. Genotyping of CYP2C19, ABCB1, PON1, PY2R12, B4GALT2, CES1, and PEAR1 was performed using Taqman^®^ Genotyping Assays. RESULTS/ANTICIPATED RESULTS: The mean PRU across the cohort was 203±61 PRU (range, 8–324), and 42 (38%) patients had HTPR. One in four individuals carried at least 1 copy of the CYP2C19*2 variant allele. Hematocrit and PON1 p.Q192R variant were inversely correlated with platelet reactivity (*p*<0.05). Multiple logistic regression showed that 27% of the total variation in PRU was explained by a history of diabetes mellitus, hematocrit, CYP2C19*2, and PON1 p.Q192R. Body mass index (OR=1.15; CI: 1.03–1.27), diabetes mellitus (OR=3.46; CI: 1.05–11.43), hematocrit (OR=0.75; CI: 0.65–0.87), and CYP2C19*2 (OR=4.44; CI: 1.21–16.20) were the only independent predictors of HTPR. DISCUSSION/SIGNIFICANCE OF IMPACT: In a representative sample of Puerto Rican patients with cardiovascular disease, diabetes mellitus, hematocrit, CYP2C19*2, and PON1 p.Q192R were associated with on-treatment platelet reactivity. These factors may identify a subset of patients at higher risk for adverse events on clopidogrel in the Hispanic population.

